# Mycoheterotrophic plants preferentially target arbuscular mycorrhizal fungi that are highly connected to autotrophic plants

**DOI:** 10.1111/nph.18310

**Published:** 2022-07-07

**Authors:** Sofia I. F. Gomes, Miguel A. Fortuna, Jordi Bascompte, Vincent S. F. T. Merckx

**Affiliations:** ^1^ Above‐Belowground Interactions Group, Institute of Biology Leiden University Sylviusweg 72 2333 BE Leiden the Netherlands; ^2^ Naturalis Biodiversity Center Darwinweg 2 2333 CR Leiden the Netherlands; ^3^ Department of Evolutionary Biology and Environmental Studies University of Zurich CH‐8057 Zurich Switzerland; ^4^ Department of Evolutionary and Population Biology, Institute for Biodiversity and Ecosystem Dynamics University of Amsterdam PO Box 94240 1090 GE Amsterdam the Netherlands

**Keywords:** antagonism, arbuscular mycorrhizal fungi, mutualism, mycoheterotrophy, mycorrhizal networks

## Abstract

- How mycoheterotrophic plants that obtain carbon and soil nutrients from fungi are integrated in the usually mutualistic arbuscular mycorrhizal networks is unknown. Here, we compare autotrophic and mycoheterotrophic plant associations with arbuscular mycorrhizal fungi and use network analysis to investigate interaction preferences in the tripartite network.
- We sequenced root tips from autotrophic and mycoheterotrophic plants to assemble the combined tripartite network between autotrophic plants, mycorrhizal fungi and mycoheterotrophic plants. We compared plant–fungi interactions between mutualistic and antagonist networks, and searched for a diamond‐like module defined by a mycoheterotrophic and an autotrophic plant interacting with the same pair of fungi to investigate whether pairs of fungi simultaneously linked to plant species from each interaction type were overrepresented throughout the network.
- Mycoheterotrophic plants as a group interacted with a subset of the fungi detected in autotrophs but are indirectly linked to all autotrophic plants, and fungi with a high overlap in autotrophic partners tended to interact with a similar set of mycoheterotrophs. Moreover, pairs of fungi sharing the same mycoheterotrophic and autotrophic plant species are overrepresented in the network.
- We hypothesise that the maintenance of antagonistic interactions is maximised by targeting well linked mutualistic fungi, thereby minimising the risk of carbon supply shortages.

## Introduction

Since the early history of life, interspecific mutualisms have been paramount in the functioning of ecosystems (Thompson, 2005; Bascompte & Jordano, 2014). Mutualisms can form complex networks of interdependence between dozens or even hundreds of species. A prime example of this ‘web of life’ is the 450‐million‐yr‐old mutualism between the majority of land plants and arbuscular mycorrhizal (AM) fungi (Strullu‐Derrien *et al*., 2018). In this interaction, plants supply the AM Glomeromycotina and Mucoromycotina fungi with carbon, essential for fungal survival and growth. In return, the fungi provide their host plants with mineral nutrients and water from the soil (Smith & Read, 2008; Bidartondo *et al*., 2011). One of the key characteristics of the AM interaction is its low interaction specificity: a mycorrhizal plant typically associates simultaneously with multiple fungi and a mycorrhizal fungus often associates simultaneously with multiple plants (Lee *et al*., 2013). This creates complex underground networks in which plants of different species are indirectly linked through shared AM fungi (Toju *et al*., 2015; Chen *et al*., 2017). Despite this low specificity, there is evidence that networks of plants and AM fungi do not assemble randomly; instead the interactions can be affected by plant functional group (Davison *et al*., 2011; Sepp *et al*., 2019), and plant or fungal evolutionary relationships (Montesinos‐Navarro *et al*., 2015; Chen *et al*., 2017).

Considerable progress has been made in recent years in dissecting the exchange of resources between plants and AM fungi and its regulation. Experimental work on low‐diversity systems has demonstrated that control is bidirectional, and partners offering the best rate of exchange are rewarded, suggesting that AM networks consist of ‘fair trade’ interactions of carbon‐for‐nutrient exchange (Bever *et al*., 2009; Kiers *et al*., 2011). However, the importance of reciprocally regulated resource exchange is questioned, as mycorrhizas also affect plant health, interactions with other soil organisms, host‐defence reactions and suppression of nonmycorrhizal competitor plants (Walder & Van Der Heijden, 2015). Also, strictly reciprocal regulation of carbon‐for‐nutrients exchange does not seem to apply to all AM interactions. For example, some exceptional plants behave as ‘cheaters’ (Selosse & Rousset, 2011; Walder & Van Der Heijden, 2015): mycoheterotrophic plants obtain carbon from root‐associated fungi and some species have replaced photosynthesis with carbon uptake from AM fungi (Leake, 1994; Merckx, 2013). Although the mechanism underpinning carbon transfer from AM fungi to mycoheterotrophic plants remains unclear, mycoheterotrophic plants are often considered cheaters of the mycorrhizal symbiosis because they have evolved from mutualistic ancestors (Merckx *et al*., 2013) and exploit the AM symbiosis for soil nutrients and carbon without reciprocating, to our current knowledge (Selosse & Rousset, 2011), or without apparently being sanctioned by the fungal partners (Walder & Van Der Heijden, 2015). Moreover, it has been suggested that mycoheterotrophic plants may display a truly biotrophic parasitic mode, digesting the fungus colonising their roots (Imhof *et al*., 2013). Despite these observations, it is unknown whether mycoheterotrophic plants have a true negative effect on their associated fungi, and we cannot rule out if they provide cryptic benefits to their symbionts.

Within obligate mutualisms, the critical barrier to mutualism breakdown and to the evolutionary stability of the resulting cheater species is thought to be a requirement for three‐species coexistence: a cheater plant relies on a mutualistic partner – a mycorrhizal fungus – which simultaneously interacts with an autotrophic plant (Pellmyr & Leebens‐Mack, 1999). In species‐rich mutualisms, such as the AM symbiosis, for which multispecies coexistence is the rule, a high potential for the occurrence of these tripartite linkages is expected (Merckx & Bidartondo, 2008). Indeed, while evolution of cheating in specialised obligate mutualisms is relatively rare (Sachs & Simms, 2006), cheating in the AM symbiosis has evolved in more than a dozen of plant clades, including over 250 species that together occur in nearly all tropical and subtropical forests (Merckx, 2013; Gomes *et al*., 2019a).

Previous work has shown that mycoheterotrophic plants target a subset of the mycorrhizal fungi available in the local community (Bidartondo *et al*., 2002; Gomes *et al*., 2017a; Sheldrake *et al*., 2017). Their associated fungal communities can vary in specificity: while many families of mycoheterotrophic plant species associate with fungi that are clustered in the Glomeromycotina phylogeny, large differences in the number of associated fungi and phylogenetic specificity are observed between species of mycoheterotrophic plants (Merckx *et al*., 2012). This specificity can be shaped by the competitive interactions of the plants. Gomes *et al*. (2017b) showed that, among mycoheterotrophs, plant species usually associate with more distantly related fungi than expected by chance, and in communities of co‐occurring mycoheterotrophic species, the phylogenetic diversity of the associated fungi increases with the extent of fungal overlap between the mycoheterotrophic species. This pattern may respond to an ecological mechanism driven by maximising co‐occurrence and avoiding competitive exclusion among mycoheterotrophic plants. However, whether partner choice of mycoheterotrophs is affected by the mutualistic interactions of their associated fungi with autotrophic plants is currently unknown.

Here, we hypothesise that mycoheterotrophic plants preferentially associate with ‘keystone’ (Mills & Doak, 1993) fungi that are well connected to many different autotrophic plants simultaneously, as these fungi are potentially more resilient to perturbations (Bascompte & Jordano, 2007) and may be the most reliable source of carbon (Waterman *et al*., 2013). In addition, as fungal traits play an important role in arbuscular mycorrhizal interactions – phylogenetically related AM fungi (assumed to have similar functional traits), preferentially interact with similar plant species (Chagnon *et al*., 2015) – we hypothesise that if partner selection in tripartite networks is trait driven we will be able to detect the influence of the phylogenetic relationships of the fungi. We tested these hypotheses on a combined tripartite mycorrhizal network of co‐occurring mycoheterotrophic and surrounding autotrophic plants linked by shared AM fungi compiled by high‐throughput DNA sequencing. To place our results in the context of recent work on comparing different types of ecological interaction networks (Melián *et al*., 2009; Fontaine *et al*., 2011; Sauve *et al*., 2013), we consider autotrophic plants to establish mutualistic interactions with AM fungi and mycoheterotrophic plants to form antagonistic interactions with AM fungi, although it remains unclear whether mycoheterotrophs have a negative impact on their associated fungi.

## Materials and Methods

### Sampling

As mycoheterotrophic plants are relatively rare and often have patchy distributions (Gomes *et al*., 2019b), we sampled two 4 × 4 m subplots a few metres apart in a coastal lowland plain rainforest in French Guiana (5°28′25″N, 53°34′51″W) on 28 July 2014, with overlapping mycoheterotrophic species. In both subplots, roots of mycoheterotrophic plants and surrounding autotrophic plants were sampled, cleaned with water, and stored on cetyltrimethylammonium bromide (CTAB) buffer at −20°C until further processing. We found, in total, five mycoheterotrophic plant species: *Dictyostega orobanchoides*, *Gymnosiphon breviflorus* (Burmanniaceae), *Voyria aphylla*, *Voyriella parviflora* (Gentianaceae) and *Soridium spruceanum* (Triuridaceae). Complete individuals of mycoheterotrophic plants were dug out and, around each, three root tips of autotrophic plants were collected. In addition, we randomly sampled five autotrophic plant root tips from each quadrant of each plot aiming to better represent the local belowground mutualistic community. In total, we collected 60 root samples of mycoheterotrophic plants and 220 autotrophic plant root samples. For the autotrophic plants, 123 samples could be identified by DNA sequencing (please refer to subsequent paragraphs). Ninety‐nine samples among 28 autotrophic, and 45 samples among the five mycoheterotrophic plant species had Glomeromycotina reads. After removing samples with < 500 Glomeromycotina reads, we retained a total of 77 samples of 21 autotrophic species and 27 samples of the five mycoheterotrophic species. A detailed list of mycoheterotrophic and autotrophic plant species collected in this study can be found in Supporting Information Table S1.

### Plant identification and fungal communities sequencing

DNA was extracted from the CTAB‐preserved roots with the NucleoMag96 Plant Kit (Macherey‐Nagel GmbH & Co., Düren, Germany), using the KingFisher Flex Magnetic Particle Processor (Thermo Fisher Scientific, Waltham, MA, USA). Autotrophic plant species were identified by sequencing the markers *matK or trnL* as described in Gomes *et al*. (2017a). Plant identification to species, genus, or family level based on Blast against GenBank was reviewed by consulting the checklist of plants in French Guiana (Funk *et al*., 2007). Fungal communities associated with the roots of mutualistic and antagonistic plants were amplified using the primers fITS7 (Ihrmark *et al*., 2012) and ITS4 (White *et al*., 1990) and sequenced using a Personal Genome Machine (Ion Torrent; Life Technologies, Guildford, CT, USA), as described in Gomes *et al*. (2017b) in two separate runs. Negative controls from the extraction and the PCR reactions were included (and had zero reads). The same bioinformatics methods from Gomes *et al*. (2017b) were used to process the raw reads from the two runs combined until clustering into 97% operational taxonomic units (OTUs), using Usearch v.7.0 (Edgar, 2010). OTUs that were represented by fewer than six reads in each sample were excluded to avoid spurious OTUs (Lindahl *et al*., 2013). The taxonomical assignment of each OTU was carried out by querying against the UNITE database (Kõljalg *et al*., 2013). Because the mycoheterotrophic plants in this study are currently only known to associate with fungi that belong to the subphylum Glomeromycotina (Merckx *et al*., 2012), we only retained fungal OTUs from this subphylum in the subsequent analysis. In these analyses, we accounted for the phylogenetic relatedness between fungal OTUs, due to the uncertainty of correspondence of individual OTUs with taxon diversity of AM fungi (Flynn *et al*., 2015), despite the fact that delimitation of OTUs is not likely to interfere with ecological interpretations (Lekberg *et al*., 2014). We inferred the phylogenetic relationships between the fungal OTUs following the strategy of Gomes *et al*. (2017b). Briefly, we aligned the OTUs with partial reference sequences and performed a phylogenetic inference in which the relationships between these references were enforced based on Krüger *et al*. (2012). From this point forwards, we refer to OTUs as ‘fungi’. The fungal communities obtained from the root samples are not necessarily similar representations of the total communities of antagonists and mutualists, because the sampling coverage of their root systems varied: mycoheterotrophic plants are small herbs, and therefore large parts of their root system were collected and extracted, whereas for autotrophic plants, mostly forest trees in our plots (see the Results section), root samples represent only a small fragment of their root system and therefore the detected fungal communities associated with autotrophic plants are likely to be a subset of their total fungal communities. To test for potential bias on the detection of fungal OTUs, due to the differences in root sampling between mycoheterotrophic and autotrophic plants, we generated accumulation curves considering both the number of reads and the individual samples for each plant type (Fig. S1). We observed that, in both cases and for both plant types, the accumulation curves tended to reach an asymptote, and therefore the potential bias of underrepresentation of fungal diversity for both plant types introduced by the uneven sampling is likely to be limited.

### Plant–fungi interactions

**Fig. 1:**
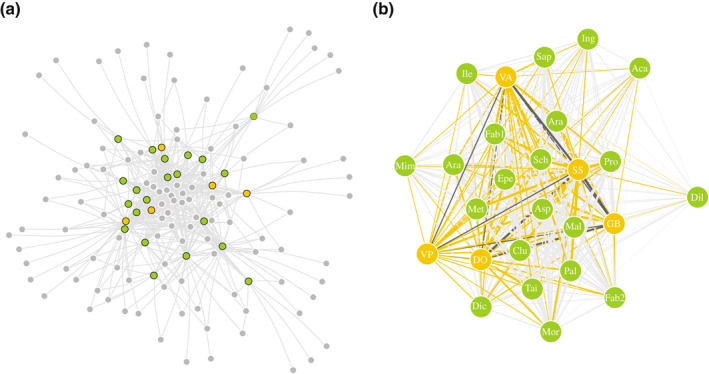
Tripartite arbuscular mycorrhizal interactions. (a) Visualisation of the tripartite network between fungi (grey) and mycoheterotrophic (yellow), and autotrophic (green) plants, in which edges represent a connection between a plant and a fungus. (b) Plant–plant–fungi overlap network, in which edges represent a link between plant species through shared arbuscular mycorrhizal fungus. The thickness of the lines represents the interaction strength between the plants (the thicker the line, the more fungi are shared). Yellow lines link mycoheterotrophic and autotrophic plants; light grey lines link autotrophic to autotrophic plants; and dark grey lines link mycoheterotrophic to mycoheterotrophic plants. Identification of autotrophic plants is indicated by the first three letters of their name (please refer to full names in Fig. 3); mycoheterotrophic plants are *Dictyostega orobanchoides* (DO), *Gymnosiphon breviflorus* (GB), *Soridium spruceanum* (SS), *Voyria aphylla* (VA), and *Voyriella parviflora* (VP). In both network representations (a, b), one of the 100 rarefied matrices to a depth of 844 reads was used; and the Fruchterman–Reingold layout was used, in which nodes are evenly distributed through the graph, in which plants that share more connections are closer to each other.

Fungal communities at the plant individual level were significantly structured by plant species identity with a minor influence of the subplot in which they were collected (Methods S1; Fig. S2). To integrate the interactions of mycoheterotrophic and autotrophic plants, we considered their plant–fungi interactions as a single tripartite network (Fig. 1a) by combining the fungal communities associated with individual plants from the two subplots into overall communities per plant species. By combining the data from the two subplots, we aimed at reconstructing a more robust picture of the interactions in this highly diverse rainforest while compensating for the relatively low sampling intensity (of 200 root tips of autotrophic plants, 123 plant species could be identified). Plant species for which Glomeromycotina fungi were represented by < 500 reads were excluded, resulting in the removal of 11 mutualistic plant species (please refer to details in Table S1). Moreover, rarefying the OTU matrix has been shown to greatly increase the false‐positive rate of OTUs per sample, which can influence the outcome of the analyses (McMurdie & Holmes, 2014). Therefore, we performed the subsequent analyses on 100 matrices rarefied to 844 reads per plant species, based on the lowest number of reads obtained for all plant species in the observed dataset. Results are presented with mean and standard deviation values obtained from running the analyses on the rarefied matrices. To investigate fungal association patterns of mycoheterotrophic and autotrophic plants, we used incidence (binary) data, because our main interest was to determine which interactions could be established and not how abundant they were. Also, due to the difference in root sampling (please refer to preceding paragraphs), read abundance was unlikely to correspond to fungal abundances at the plant species level, in particular between plant types (mycoheterotrophs and autotrophs). We considered the simultaneous presence of a particular fungus in the roots of a mycoheterotrophic and an autotrophic as a potential link between both (Southworth *et al*., 2005).

We tested for the effect of plant and fungi phylogenetic relatedness on the observed interactions in both networks. We used the fungal phylogeny described above, and the phylogenetic relationships of the plants as derived from TimeTree (Kumar *et al*., 2017) (please refer to details in Methods S2). We computed Mantel test correlations between the phylogenetic distance matrix and the community dissimilarity matrix in each instance for autotrophic and mycoheterotrophic species individually using the vegan R package (Oksanen *et al*., 2015). The phylogenetic distance matrices were extracted from the phylogenetic trees of plants and fungi, and the community dissimilarity matrices were calculated as the Jaccard distance on the binary interaction matrices. Phylogenetic signal was calculated for the 100 rarefied matrices, and consistency of significant results was assessed across multiple rarefaction depths (Fig. S3).

### Mutualistic and antagonistic plant–plant interactions

To compare the range of fungal interactions between autotrophic and mycoheterotrophic plants, we calculated their normalised degree and the phylogenetic species variability of their associated fungal communities. The normalised degree of a plant species is the proportion of its associated fungi out of the total possible fungi in the network (Martín González *et al*., 2010), and was calculated with the *ND* function of the bipartite R package (Dormann *et al*., 2018). The phylogenetic species variability (*psv*) of the fungal community associated with a plant species summarises the level to which the fungi in this community are phylogenetically related (Helmus *et al*., 2007). When a community consists of unrelated fungi, the index equals 1, indicating maximum phylogenetic variability. As relatedness increases, the index approaches 0, indicating high phylogenetic specificity (Helmus *et al*., 2007). In addition, we calculated the fungal overlap between each pair of plant species as the number of fungi they share to infer plant–plant interactions (Fig. 1b).

### Mutualistic and antagonistic fungal interactions

We compared the ecological similarity of the fungi shared between the two interaction type networks (i.e. mutualistic and antagonistic interactions). The ecological similarity of a pair of fungi represents their similarity in interactions through shared plants. We calculated similarity matrices between the fungi linked to autotrophic and mycoheterotrophic plants separately, using two different measures. The first measure is the Jaccard index, which corresponds to the number of plant species with which both fungi interact divided by the total number of plant species with which they interact, and the second is the overlap measure: *C*_*ij*_/min(*d*_*i*_, *d*_*j*_), where *C*_*ij*_ is the number of shared plants between fungus *i* and *j*, and min(*d*_*i*_, *d*_*j*_) is the smallest number of associated plants between fungus *i* and *j* (Saavedra *et al*., 2013). The overlap measure corresponds to the number of shared plant species relative to the maximum number of plant species that can be shared, while taking into account the possibility of fungi having differential limits in their maximum number of plant partners. We computed the Mantel test correlation between the similarity matrices for the mutualistic and antagonistic interactions using the two different measures. We also computed the partial Mantel test correlation between the similarity matrices calculated using the Jaccard and overlap measures, controlling for the phylogenetic relatedness of the shared fungi.

### Network analysis

**Fig. 2:**
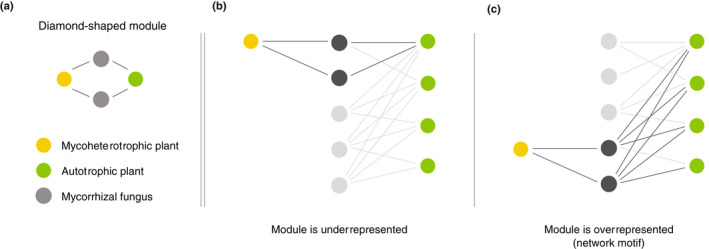
Diamond‐shaped module investigated in this paper. Representation of the module linking pairs of fungi to the same mycoheterotrophic and autotrophic plant species (a). Examples of underrepresentation (b) and overrepresentation (c) of the module in a network. When the module is overrepresented it is considered a network motif.

To investigate how mycoheterotrophic plants are integrated in the mutualistic network of fungi and autotrophic plants, we searched for diamond‐shaped modules in the tripartite network, representing an autotrophic and a mycoheterotrophic plant interacting with the same pair of fungi (Fig. 2a). If the module is overrepresented throughout the entire network, it can be considered a *motif* (Milo *et al*., 2002; Bascompte & Melián, 2005; Stouffer *et al*., 2007). This specific motif does not consider the identity of the partners *per se*, but searches for this particular topological structure. Because our network consists of a tri‐trophic ‘food chain’ (considering the exchange of carbon), we built on the existing theoretical knowledge of trophic interrelationships and selected the diamond‐shaped module among those that were part of the theoretical research agenda on food webs. This module was described as ‘apparent competition’ and has been found to be overrepresented, and therefore highly relevant, in real food webs (Milo *et al*., 2002; Bascompte & Melián, 2005). Although the importance of competition in AM interactions is unclear, we expect this module to be effective for testing our main hypothesis: if mycoheterotrophic plant species associate with fungi that are linked to only few autotrophic plant species, the module will be underrepresented (Fig. 2b). By contrast, if mycoheterotrophic plant species associate with fungi that are well connected to autotrophic plants, many of these fungi will be linked to the same autotrophic plant species and therefore the module will be overrepresented throughout the entire network (Fig. 2c) and can be considered a network motif (Milo *et al*., 2002; Bascompte & Melián, 2005; Stouffer *et al*., 2007). We did not consider alternative modules, which are potentially also relevant in this context, but this an interesting follow‐up to the present paper.

To assess whether the diamond‐shaped module was overrepresented relative to random expectations, we randomised the original mycoheterotrophic plants – mycorrhizal fungi – autotrophic plants community matrix and calculated the frequency of this diamond‐shaped module in 1000 generated random networks. We used a null model that draws an interaction between a plant and a fungal species that is proportional to the generalisation level of both species. Specifically, this probability is defined as the arithmetic mean of the fraction of interactions of the plant and that of the fungi (null model 2 in Fortuna & Bascompte, 2006; initially proposed in Bascompte *et al*., 2003). The null models were constructed with two different sets of fungi. First, we generated null models using all fungi in the network. This null model tests whether mycoheterotrophic plants exhibit an overall preference for fungi that are well connected to the autotrophic plants among all fungi present. Next, we generated null models only using fungi that are shared between mycoheterotrophic plants and autotrophic plants in the empirical network. This null model is similar to the previous model, but by restricting the resampling only to overlapping fungi between mycoheterotrophic and autotrophic plants, we assess whether there is a further preference for well connected fungi among those used by the mycoheterotrophs. Because there was an imbalanced number of plant species (five mycoheterotrophic and 21 autotrophic plants), each randomised matrix resulted from randomising the antagonistic and mutualistic interactions separately, and then were combined into a single matrix before the module search. To assess the potential effects of sampling effort both in the number of individuals collected (leading to roughly half of fungal reads belonging to the five mycoheterotrophic species, whereas the other half represent 21 autotrophic species) and in representation of whole (mycoheterotrophic) or partial (autotrophic) root systems, we repeated this procedure for a set of 100 rarefied original matrices. Furthermore, as plant species are represented by an unequal number of samples (Table S1), we also repeated the module search on 1000 matrices created by random resampling three samples per plant species (discarding species for which fewer than three samples were available). When this procedure showed that the number of individuals per species affected the consistency of the results, we alternatively tested the robustness of sampling by repeating the module search at multiple read depths in the rarefaction step to exclude an effect of the chosen sampling depth (please refer to preceding paragraphs). The strategy of resampling only three individuals per species reduced the representation of autotrophs in relation to mycoheterotrophs, from a proportion of 4 : 1 to 2 : 1, drastically deviating from the empirical situation. However, to the best of our knowledge, there was no alternative way to address the potential effect of the sampling bias in our study.

To evaluate whether the diamond‐shaped module was overrepresented throughout the empirical network, we calculated the standard $z‐\text{score}=\left(\mathrm{obs}\;-\;\bar{\mathrm{rand}}\right)/\mathrm{SD}\left(\mathrm{rand}\right)$, where *obs* is the observed network modules and $\bar{\mathrm{rand}}$ is the mean of the same type of modules across the 1000 random networks using a 95% confidence interval. An empirical result above the null confidence interval indicates that pairs of fungi are shared between particular mycoheterotrophic and autotrophic plants more often than expected by chance, reflecting a preference of mycoheterotrophic plants to link to pairs of fungi that are generalists in their interactions with autotrophic plants. An empirical result below the null confidence interval indicates that mycoheterotrophic plants target pairs of fungi that are specialists in their interactions with autotrophic plants.

## Results

### Plant–fungi interactions

We successfully identified root tips of 32 autotrophic (123 root tips) and five mycoheterotrophic (60 individuals) plant species (please refer to Table S1 for detailed species list). After retaining samples that contained a minimum of 500 reads identified as Glomeromycotina, we reduced our dataset to 21 autotrophic (77 root tips) and five mycoheterotrophic (27 individuals) plant species, with a total of 365 135 reads. Autotrophic plants belonged to 14 families, and included 16 trees, two shrubs, one herb, one vine and one liana. We obtained 115 AM fungi, identified as Glomeromycotina within three families Gigasporaceae (four fungi), Acaulosporaceae (14) and Glomeraceae (97). Of these, 96 OTUs (49% of total reads) were present in the antagonistic network.

The 100 rarefied binary matrices included 26 plant species (21 autotrophs and five mycoheterotrophs) and 110 ± 1.47 fungi. Of these, mutualistic networks had 100 ± 1.51 fungi (reduced from 115 fungi before rarefaction) and antagonistic networks had 61 ± 3.14 fungi (reduced from 96 fungi before rarefaction). In all the generated matrices, autotrophic and mycoheterotrophic plants shared 51 ± 3.16 fungi, which represents 55% and 92% of total fungi present in autotrophic and mycoheterotrophic, respectively (Fig. 1a).

We measured a relatively low but significant phylogenetic signal of the fungal phylogeny on the antagonistic (*r* = 0.20 ± 0.06, *P* = 0.003 ± 0.005) and mutualistic (*r* = 0.21, *P* = 0.001) networks (please refer to the effect of rarefaction depth on phylogenetic signal in Fig. S3). We found no significant effects of plant phylogeny in any of the networks.

### Mutualistic and antagonistic plant–plant interactions

**Fig. 3:**
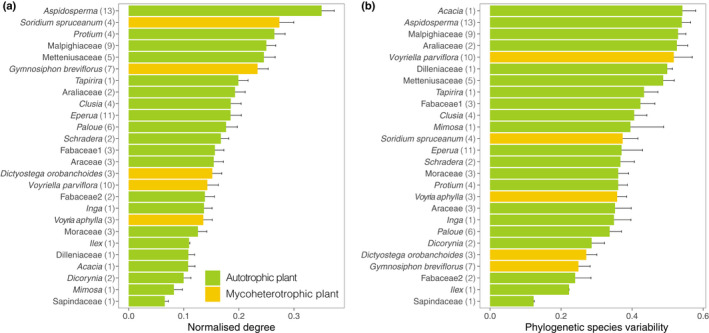
Mean ± SD plant normalised degree (a) and phylogenetic species variability (b) for the 21 autotrophic and five mycoheterotrophic plant species, resulted from the 100 rarefactions to a depth of 844 reads. Values between parentheses represent the number of individual samples per species.

The number of fungal partners per plant species varied, with *Aspidosperma* sp. showing the highest normalised degree and Sapindaceae the lowest amongst the autotrophic plants (Fig. 3a). Ranked over all the plants, three mycoheterotrophic species, namely *V. aphylla*, *V. parviflora* and *D. orobanchoides*, presented a normalised degree in the lower half of the spectrum, whereas *G. breviflorus* and *S. spruceanum* are amongst the plants with the highest number of associated fungi. We also observed a wide range of phylogenetic species variability (*psv*) for autotrophic plants, in which *Acacia* sp. and *Aspidosperma* sp. had the highest *psv* and Sapindaceae the lowest, throughout which, the mycoheterotorphic plants are distributed (Fig. 3b).

Among the mycoheterotrophic plants, we observed that *S. spruceanum* shared most fungal interactions with *G. breviflorus*, followed by *S. spruceanum* with *D. orobanchoides* and *V. aphylla*, and also *G. breviflorus* with *D. orobanchoides*. We observed that mycoheterotrophic plants as a group associate with fungi that are simultaneously linked to all autotrophic plants that were retained in our analyses (Fig. 1b). Of all the possible connections between mycoheterotrophic and autotrophic plant species, *S. spruceanum* had the highest fungal overlap with *Clusia* sp., and then with *Tapirira* sp. and *Protium* sp.; *G. breviflorus* with *Aspidosperma* sp. and *Protium* sp., then with *Schadera* sp. and *Clusia* sp.; *D. orobanchoides* with *Tapirira* sp. and then with Fabaceae sp. 1; *V. parviflora* with *Aspidosperma* sp. and then with Araceae sp., Malpighiaceae sp. and *Clusia* sp; and *V. aphylla* with *Aspidosperma* sp. and then with *Paloue* sp. These autotrophic plants are also those with highest number of fungal interactions overall (Fig. 3a). Among the autotrophic plants, *Aspidosperma* sp. had the highest fungal overlap with *Protium* sp., Malpighiaceae sp. and Metteniusaceae sp., which are also among the highest ranked species in terms of number of associated fungi and phylogenetic diversity (Fig. 3).

### Mutualistic and antagonistic plant–fungi interactions

**Fig. 4:**
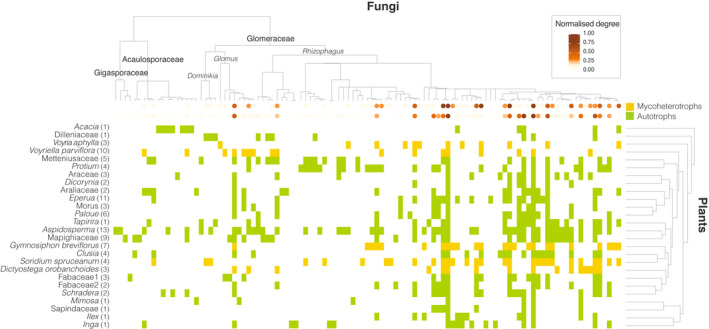
Plant–fungi interactions of autotrophic (green) and mycoheterotrophic (yellow) plants using a matrix rarefied to a depth of 844 reads. Phylogenetic relationships between the fungi are shown at the top. Plant species are listed on the left with the number of individual samples in between parentheses; hierarchy clustered dendrogram based on the Bray–Curtis distance of their fungal communities is shown on the right. The intensity of the orange dots on the tips of the fungal phylogeny depicts the normalised degree (representing their interaction strength) of each fungus in each of the plant–fungi networks (i.e. in the mycoheterotrophic plants–fungi and autotrophic plants–fungi networks). Values between parentheses represent the number of individual samples per species.

Fungi that present in the mutualistic network but are absent from the antagonistic network were generally characterised by a low normalised degree, except for two *Rhizophagus* taxa that were present in the majority of autotrophic plants (Fig. 4). Moreover, we found a significant correlation of fungal ecological similarity between the mutualistic and antagonistic interactions (Mantel tests: Jaccard *r* = 0.20 ± 0.04, *P* = 0.002 ± 0.002; overlap *r* = 0.24 ± 0.04, *P* = 0.001), and also when accounting for the phylogenetic signal of the fungi (partial Mantel tests: Jaccard *r* = 0.18 ± 0.003, *P* = 0.018 ± 0.04; overlap *r* = 0.22 ± 0.04, *P* = 0.002 ± 0.003). We also observed that the fungi with the highest normalised degrees, both in the mutualistic and antagonistic network, were all members of the Glomeraceae family (Fig. 4).

### Network analysis

The network analysis indicated the presence of 2620.53 ± 271.23 instances of the diamond‐shaped module in the empirical networks across the rarefied matrices. The module was overrepresented in relation to random expectations for all the 100 rarefied matrices, both when including all the fungi in the dataset (*z*‐score = 7.88 ± 1.06, *P* = 0), and also when pruning the dataset to only include the overlapping fungi (*z*‐score = 3.43 ± 0.45, *P* = 0.003 ± 0.006). Repeating the analysis on resampled matrices that drew three random individuals per species resulted in an overrepresentation of the module in 99% of the cases when including all the fungi in the dataset (modules: 1129.17 ± 280.98, *z*‐score = 3.00 ± 0.49, *P* = 0.007 ± 0.010), and in an overrepresentation of the module in 24.4% of the cases when including only the overlapping fungi in the dataset (modules: 1164.29 ± 305.39, *z*‐score = 1.49 ± 0.42, *P* = 0.089 ± 0.060). Using the overlapping fungi dataset, the repeated analysis for multiple rarefaction depths indicates that, despite the number of diamond‐shaped modules increases with increasing rarefaction depth, it does not affect the result that pairs of fungi share a mycoheterotrophic and an autotrophic plant more often than expected by chance (Fig. S4).

## Discussion

We found that mycoheterotrophic plants as a group target with a subset of the fungi that are potentially available, however this subset of fungi is associated with all autotrophic plants detected in this study. The results of the network analysis indicate that this pattern is produced by a preference of the mycoheterotrophs for well connected fungi. Therefore, despite associating only with a subset of the local pool of fungi, the mycoheterotrophs indirectly reach a wide range of autotrophic plants through their shared fungi, potentially obtaining carbon from any of the autotrophic plants at the study site. Fungi not detected in the roots of mycoheterotrophs were generally connected to few autotrophic plants. Within the fungi that are shared between the mutualistic and antagonistic networks, we detected a significant ecological symmetry between the mutualistic and antagonistic interactions of the fungi: pairs of fungi that interact with overlapping sets of autotrophic plants also interact with overlapping mycoheterotrophic plants. The network analysis indicated that this pattern occurs more often than expected by chance. Based on the highest fungal overlap between each mycoheterotrophic species with different subsets of highly connected autotrophic species, this pattern appears to be driven by a further preference of mycoheterotrophs for fungi that are well linked to specific mutualistic plants. These autotrophic plants are the ultimate sources of the carbon that mycoheterotrophic plants take up from the fungi shared between autotrophs and mycoheterotrophs. Therefore, we suggest that the observed pattern reflects a strategy in which the maintenance of antagonistic interactions is maximised by targeting well linked fungi, thereby minimising the risk of carbon supply shortages.

### Fungal preferences of mycoheterotrophic plants

We found that plant species identity had a significant influence on the fungal community composition, regardless of the plant type, which indicates that these communities are nonrandom subsets of the local fungal taxon pool. This supports previous evidence that co‐occurring plant species showed differences in selectivity towards available AM fungi (Davison *et al*., 2011). Mycoheterotrophic plant species are known to select particular groups of fungi, often a narrower range than the surrounding autotrophic plants (Bidartondo *et al*., 2002; Gomes *et al*., 2017a,b). Here we observed that five co‐occurring mycoheterotrophic plant species collectively associate with approximately half of the available fungal taxa (Fig. 4). Antagonistic interactions can therefore be supported by a relatively wide array of AM taxa, as shown previously (Merckx *et al*., 2012; Gomes *et al*., 2017b; Sheldrake *et al*., 2017). Although fungi from three different fungal families (Glomeraceae, Acaulosporaceae and Gigasporaceae) were detected in the roots of mycoheterotrophic plants, a clear preference for Glomeraceae taxa, and *Rhizophagus irregularis* relatives in particular, was observed. The taxa of this clade were the most frequently encountered in the roots of the autotrophic plants as well (Fig. 4). *Rhizophagus* contains some of the most globally widespread and common AM fungi (Kivlin *et al*., 2011; Moora *et al*., 2011; Davison *et al*., 2015; Gomes *et al*., 2018) although this finding is mostly derived from studies in temperate areas. Our results indicate that the tropical rainforest offers no exception to this pattern. Glomeraceae are usually not only the most dominant clade in natural AM communities, often accounting for *c*. 70% of all species (Montesinos‐Navarro *et al*., 2012), but they also have been found consistently to include the most generalist AM fungi in other network studies (Montesinos‐Navarro *et al*., 2012; Chagnon *et al*., 2015; Chen *et al*., 2017). The ability to interact with many autotrophic plant species can be a potential reason for why mycoheterotrophic plants generally target Glomeraceae fungi (Merckx *et al*., 2012; Renny *et al*., 2017). Ecological theory predicts that generalist species tend to have large distribution ranges (Brown, 1984) and, consequently, are less vulnerable to (local) extinction than specialised species (Schleuning *et al*., 2016). Therefore, associations with generalist fungi may be advantageous for the evolutionary persistence of mycoheterotrophs. Furthermore, associations with multiple autotrophic plant partners may increase fungal resilience to disturbance, while mediating temporal fluctuations in carbon flow and interaction dynamics (Bennett *et al*., 2013). Therefore, this would guarantee a continuous carbon supply to the entire network without the pronounced negative effects, even in the presence of antagonists. In addition, in the context of mycorrhizal fungi, which can be linked to different plant species simultaneously (Montesinos‐Navarro *et al*., 2012), generalist fungi are therefore likely to be more reliable carbon sources for mycoheterotrophs. An alternative and perhaps not mutually exclusive explanation for why mycoheterotrophic plants preferentially target well connected fungi may be that these fungi are less effective in detecting and excluding nonphotosynthetic plant partners (Bruns *et al*., 2002; Egger & Hibbett, 2004; Bidartondo, 2005; Walder & Van Der Heijden, 2015). In contrast with our local‐scale study, Perez‐Lamarque *et al*. (2020), who performed a global‐scale study on AM mycoheterotrophic plants, reported that these plants tended to interact with (globally) specialised fungi. Although their results may have been influenced by the limited availability of global data – data of less than *c*. 0.2% of all autotrophic AM plants were available – it is possible that the most‐connected fungi in our study are less well connected in other habitats. In this case, the pattern of global‐scale reciprocal specialisation between mycoheterotrophs and AM fungi might be influenced by the specific local environmental conditions under which mycoheterotrophy occurs, such as low soil fertility (Gomes *et al*., 2019b).

### Fungal links between mutualistic and antagonistic networks

We detected that pairs of fungi that interact with similar sets of autotrophic plants share links with overlapping mycoheterotrophic plants. Therefore, there is a high level of interaction symmetry between mutualistic and antagonistic mycorrhizal networks. Also, we measured a significant influence of the fungal phylogenetic relationships on both the mutualistic and antagonistic interactions, showing that closely related fungi interact with similar autotrophic and mycoheterotrophic plants respectively. Because biotic interactions are mediated by functional traits, and most functional traits are evolutionarily conserved, a shared evolutionary history of fungi can serve as a proxy for functional similarity (Chagnon *et al*., 2015). We therefore hypothesise that both mutualistic and antagonistic interactions are shaped partially by evolutionary conserved functional traits of the fungi. In this case, the apparent preference for members of the Glomeraceae family may indicate a higher reliance on ruderal AM fungi (Chagnon *et al*., 2013) for both autotrophic and mycoheterotrophic plants. Moreover, multiple clades within this family seem to be preferentially associated with mycoheterotrophic plants, which could reflect more fine discrimination of traits that we cannot discern with the current knowledge on AM fungal strategies. In addition, the network analysis indicated that pairs of fungi shared a mycoheterotrophic and an autotrophic plant more often than expected by chance. This analysis solely indicates that the diamond‐shaped module is overrepresented in the empirical network (Fig. 2), without reference to species degree or species identity. However, our results support the idea that the observed pattern is driven by the tendency of mycoheterotrophic plants to target fungi that are well linked to autotrophic plants (Fig. 1b). The autotrophic plants with the highest fungal overlap in relation to the mycoheterotrophic plants are among those with the highest ranked degree and phylogenetic species variability from the pool of detected autotrophic plant species (Fig. 3). Moreover, fungi with the highest number of interactions in the mutualistic network are also among the best connected fungi in the antagonistic network (Fig. 4). Our findings therefore reveal that mycoheterotrophic plants preferentially associate with fungi that are simultaneously linked to a wide range of autotrophic plants. Targeting well connected fungi in the mutualistic network could be a strategy for mycoheterotrophic plants to increase their resistant and resilient facing perturbations. Although many mycoheterotrophic plants share a large number of fungi with *Aspidosperma* sp., which has the highest normalised degree among the autotrophic plants, mycoheterotrophic plant species also indirectly associate with nonoverlapping sets of autotrophic plants, as indicated by their divergent positions in the plant–plant interaction network (Fig. 2b). Therefore, building on the hypothesis that mycoheterotrophic plants maximise their coexistence by increasing the phylogenetic diversity of the AM fungi with which they associate as the overlap among co‐occurring species increases (Gomes *et al*., 2017b), the present study suggests that the differential preference of mycoheterotrophic species for connections with nonoverlapping autotrophic species may contribute to competition avoidance among mycoheterotrophic plants.

### Potential sampling biases

Considering that rainforests are species‐rich ecosystems (ter Steege *et al*., 2013), it is likely that, despite our efforts, the sampling of the belowground diversity of AM fungi, and their plant partners remained incomplete, in part because both plant identity and fungal communities of only 35% of autotrophic plants samples could be obtained. Therefore, not all autotrophic plant species present at the sites were included in the network and the reported patterns must be interpreted with caution. However, the large fungal overlap between the mutualistic and antagonistic networks suggested that it is unlikely that exclusive fungal connections of mycoheterotrophs to nonrepresented autotrophic plants are prevalent.

Furthermore, the representation of roots from mycoheterotrophic and autotrophic plants was necessarily imbalanced, as whole and partial root systems, respectively, were collected. This probably has an impact on the completeness of the fungal communities of the autotrophic plant species, and made the use of read abundances to estimate interaction strengths impossible. Moreover, the choice of primer set can introduce biases in the discovery of fungal diversity (Lekberg *et al*., 2018). We used the fITS7/ITS4 primer pair to characterise the fungal communities associated with the plants in our study, and found that mycoheterotrophic plants were associated primarily with fungi in the Glomeraceae family, which agrees with previous studies that used the SSU region (Merckx *et al*., 2012; Renny *et al*., 2017). Glomeraceae fungi have also been revealed to predominate in roots of autotrophic plant species (Davison *et al*., 2015).

Importantly, previous studies have highlighted the importance of sampling intensity (i.e. the number of possible interactions per node, which directly impacts the normalised degree per species) on network metrics (Blüthgen *et al*., 2007; Dormann *et al*., 2009). As the plant species in our study are represented by different numbers of samples, we assessed carefully any potential impacts of sampling bias on the results of the network analysis using multiple strategies. First, we randomised the mycoheterotrophic and autotrophic matrices to build the null models separately as the ratio of autotrophic and mycoheterotrophic species was only 4 : 1. Second, we calculated the number of modules over 100 rarefied matrices (which greatly reduced the number of discovered fungi for the mycoheterotrophic plants) and null models showed the presence of a network motif in 100% of the cases. Third, as the number of samples per species was also unequal across plant species, we repeated the module search procedure with random resampling of three individual samples per species and while discarding species for which fewer than three samples were available (even though this led to an unrealistic proportion of mycoheterotrophic to autotrophic plant species). This approach showed that the overrepresentation of the module in the network with all fungi was not influenced by unequal sampling across plant species. For the network with overlapping fungi, the empirical overrepresentation of the diamond‐shaped module was potentially influenced by unequal sampling, therefore we also verified the consistency of this result across multiple rarefactions depths by the use of multiple rarefied matrices at each depth (Gotelli & Colwell, 2001).

While we acknowledge that the completeness of sampling in our study is not ideal – a typical challenge for any study on mycorrhizal diversity – we also considered several factors that allowed us to separate the statistical patterns in our data from the influence of sampling effort, both in terms of the plant species detected in the sampled roots, and in their potentially incomplete number of fungal associations. Whether our results are potentially influenced by spatial patterns in the distribution of autotrophs, mycoheterotrophs and fungi remains to be determined.

### Conclusions and future perspectives

To our knowledge, our study is the first to assess how mycoheterotrophic plant species are embedded in mutualistic mycorrhizal networks. We found that mycoheterotrophic plants as a group interacted with a subset of the available fungal partners, and generally targeted fungi that were well connected to autotrophic plants. Although mycoheterotrophic species show overlap in their fungal associations, we found that they were indirectly linked to different sets of autotrophic plants, suggesting a potential mechanism to avoid competition by preferentially relying on different carbon sources (Gomes *et al*., 2017b). The phylogenetic relationships between the fungi, probably a proxy for fungal traits, had a significant influence on these nonrandom tripartite interactions. Therefore, we concluded that the persistence of mycoheterotrophs in AM networks is dependent on particular well connected ‘keystone’ mycorrhizal fungi, which provide the mycoheterotrophs with carbon from a wide range of plants. Our observations that fungi connected mutualistic and antagonistic networks in a nonrandom fashion and that well connect fungal nodes in AM networks were more prone to be targeted by mycoheterotrophs, are similar to those of Sauve *et al*. (2016) for a plant–pollinator–herbivore network when considering binary interactions. Further research is needed to assess whether this is a general feature of interactions within species‐rich communities, also when taking interaction strength into account. Our study emphasises the raising of awareness of considering multiple interaction types simultaneously (e.g. antagonistic and mutualistic) to deepen our understanding of complex biodiversity patterns (Losapio *et al*., 2021).

In contrast with ectomycorrhizal symbiosis, for which it has been known for decades that several plant species are able to combine photosynthesis and carbon uptake from fungi, in a strategy termed ‘partial mycoheterotrophy’ (Selosse & Roy, 2009), only recently this mode of life has been suggested to be widespread within the AM symbiosis. Giesemann *et al*. (2021) have shown that many photosynthetic understory plants are potentially able to take up carbon from associated AM fungi. Future work will enlighten us on whether these partially mycoheterotrophic plants rely on similar sets of fungi and rely on similar interaction patterns within the mycorrhizal network as the fully mycoheterotrophic plants in in the present study.

## Author contributions

All authors designed the study; SIFG and VSFTM collected the materials; SIFG performed the laboratory work and data analysis with input from MAF, JB and VSFTM authors. SIFG, MAF, JB and VSFTM wrote the manuscript.

## Supporting information


**Fig. S1** Accumulation curves considering the cumulative number of reads and the cumulative number of samples for autotrophic and mycoheterotrophic plants.
**Fig. S2** Venn diagrams representing variation explained by plant species identity, plant type and subplot.
**Fig. S3** Phylogenetic signal analysis repeated on multiple rarefaction depths.
**Fig. S4** Motif analysis repeated on multiple rarefaction depths.
**Methods S1** Effect of plant identity, plant type and subplot on the structure of fungal communities at the plant individual level.
**Methods S2** Plant root identification.
**Table S1** Plant identity of autotrophic and mycoheterotrophic plants from this study.
Please note: Wiley Blackwell are not responsible for the content or functionality of any Supporting Information supplied by the authors. Any queries (other than missing material) should be directed to the *New Phytologist* Central Office.

## Data Availability

The raw data that support the findings of this study are openly available in the Short Read Archive under project no. PRJNA846290.
