# Supplementary material for: Mycoheterotrophic plants preferentially target arbuscular mycorrhizal fungi that are highly connected to autotrophic plants
Source: New Phytol. 2022 Jul 7;235(5):2034–45. doi: 10.1111/nph.18310 (PMC9539982; doi:10.1111/nph.18310)
Supplement: Supplementary file 1 — Fig. S1 Accumulation curves considering the cumulative number of reads and the cumulative number of samples for autotrophic and mycoheterotrophic plants. Fig. S2 Venn diagrams representing variation explained by plant species identity, plant type and subplot. Fig. S3 Phylogenetic signal analysis repeated on multiple rarefaction depths. Fig. S4 Motif analysis repeated on multiple rarefaction depths. Methods S1 Effect of plant identity, plant type and subplot on the structure of fungal communities at the plant individual level. Methods S2 Plant root identification. Table S1 Plant identity of autotrophic and mycoheterotrophic plants from this study. Please note: Wiley Blackwell are not responsible for the content or functionality of any Supporting Information supplied by the authors. Any queries (other than missing material) should be directed to the New Phytologist Central Office. [file NPH-235-2034-s001.pdf]

New Phytologist

New Phytologist Supporting Information

Article title: Mycoheterotrophic plants preferentially target arbuscular mycorrhizal fungi that are highly connected to autotrophic plants
Authors: Sofia IF Gomes, Miguel A Fortuna, Jordi Bascompte, Vincent SFT Merckx
Article acceptance date: 19 April 2022

The following Supporting Information is available for this article:

Methods S1| Effect of plant identity, plant type and subplot on the structure of fungal communities at the plant individual level.

Methods S2 | Plant root identification.

Fig S1 | Accumulation curves considering the cumulative number of reads (top panels) and the cumulative number of samples (bottom panel) for autotrophic (left panels) and mycoheterotrophic (right panels) plants.

Fig S2 | Venn diagrams representing variation explained by plant species identity, plant type and subplot.

Fig S3 | Phylogenetic signal analysis repeated on multiple rarefaction depths.

Fig S4 | Motif analysis repeated on multiple rarefaction depths.

Table S1 | Plant identity of autotrophic and mycoheterotrophic plants from this study.

Methods S1 | Effect of plant identity, plant type and subplot on the structure of fungal communities at the plant individual level:

We explored the effects of plant identity, plant type (antagonist vs mutualist) and subplot origin on the fungal community composition obtained from the roots, at the individual plant level. For that, we rarefied each individual sample to 100 reads, and calculated the variance explained by these three factors using the function *varpart* using the *vegan* (Oksanen *et al.*, 2015) R package. We used two distance matrices by calculating the Bray-Curtis dissimilarities on the Hellinger transformed rarefied counts, and the Unifrac distance (Lozupone & Knight, 2005) to account for fungal relatedness. The variation partitioning analysis revealed that plant species identity captured most of the variation in the data, including the distinction between plant types (antagonist and mutualist), with a minor proportion of the variance explained by subplot (see Supporting Information, Figure S2), which can be expected due to the high heterogeneity of microbial communities in the soil at the local scale (Jacquemyn *et al.*, 2014). Then, to test for significance of association, we built a distance-based redundancy analysis (db-RDA) model for each matrix with these three factors, and performed model selection with the *ordi2step* function. The best model based showed that fungal communities were significantly structured by plant species identity, both considering their shared interactions only (F = 1.848, P = 0.001), or simultaneously accounting for the phylogenetic relationships between the shared fungi (F = 3.015, P = 0.001), explaining 44.8% or 57.7% of the variance in the data, respectively. The effect of plant type and plot were not significant in the db-RDA model.

Methods S2 | Plant root identification:

Plant roots were identified to genus or family level based on *matK* or *trnL* sequencing using BLAST. The genus of the BLAST hit with the highest percent identity to the query sequence was used as the genus identification, unless this genus is not known to occur in the region. Query sequences with a similarity of 99% or higher were considered to belong to the same plant species. A time tree was constructed based on genus identification using TimeTree.org. For groups sequences that could not be identified to genus level with certainty, the genus of the best BLAST hit was used.

Fig S1 | Accumulation curves considering the cumulative number of reads (top panels) and the cumulative number of samples (bottom panel) for autotrophic (left panels) and mycoheterotrophic (right panels) plants.

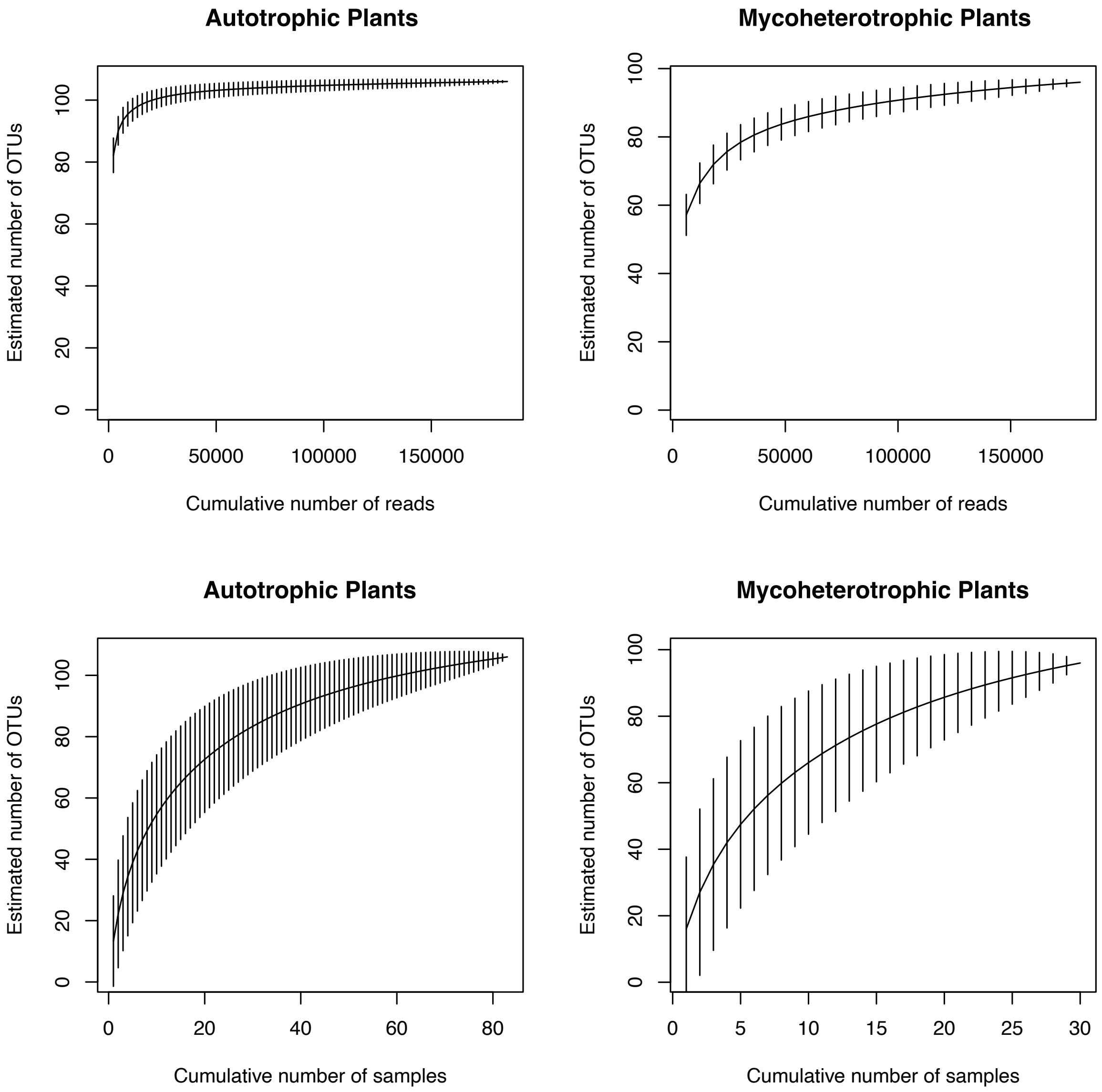

Fig S2 | Venn diagrams representing variation explained by plant species identity, plant type and subplot using variation partitioning.

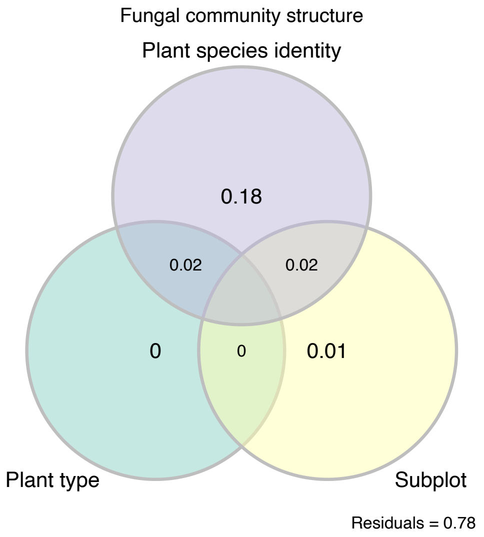

Fig S3 | Phylogenetic signal analysis repeated on multiple rarefaction depths. Mean observed phylogenetic signal of fungi in the antagonistic (top) and mutualistic (bottom) networks for the 100 rarefaction matrices tested per rarefaction depth (left), and corresponding mean p-values (right). Bars represent standard deviation.

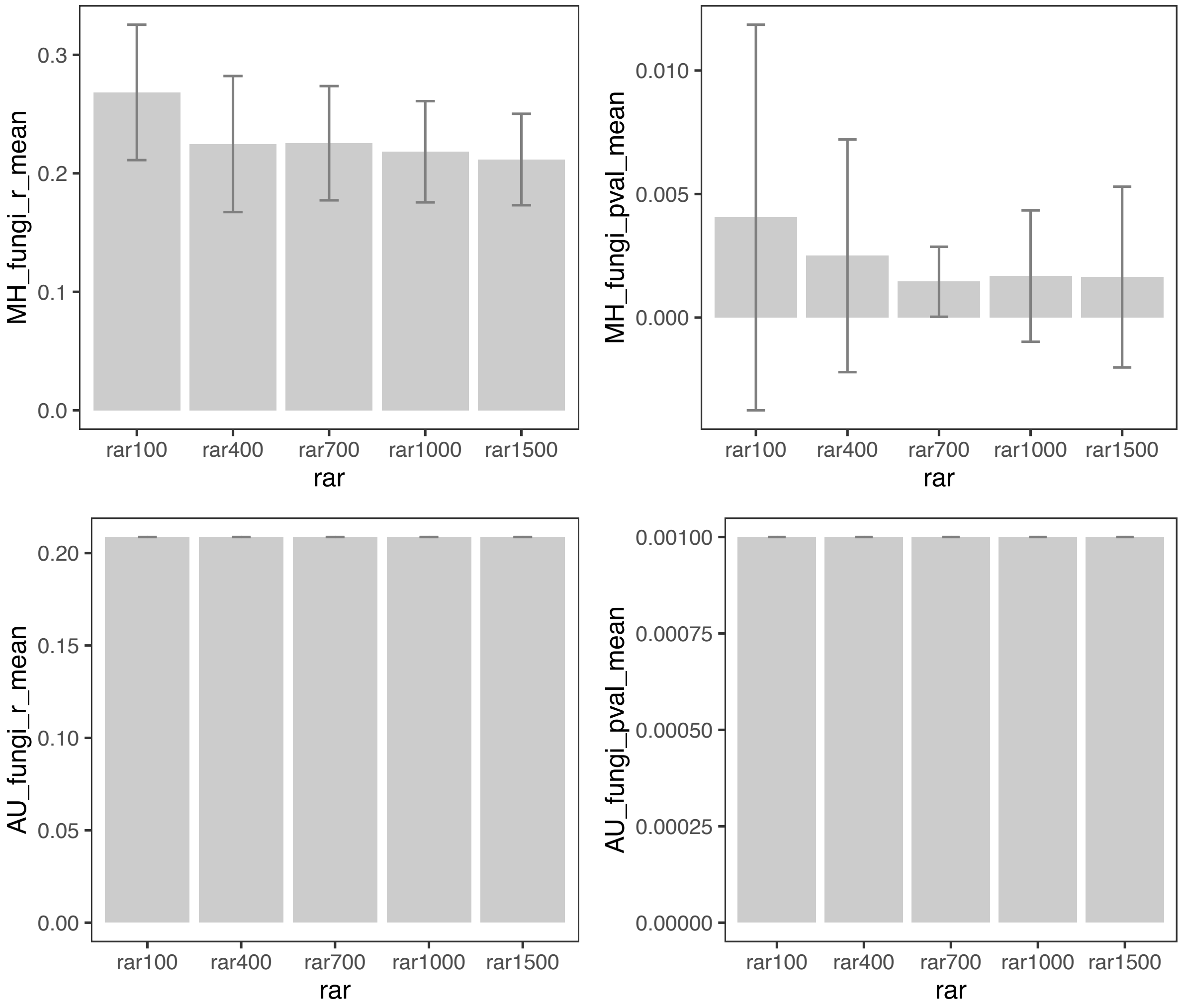

Fig S4 | Motif analysis repeated on multiple rarefaction depths. Mean observed motifs for the 100 rarefaction matrices tested per rarefaction depth (left), and corresponding mean z-scores (right). Dashed lines represent the critical z-score values for 95% (y = 1.96) and 99% (y = 2.58) confidence levels. Bars represent standard deviation.

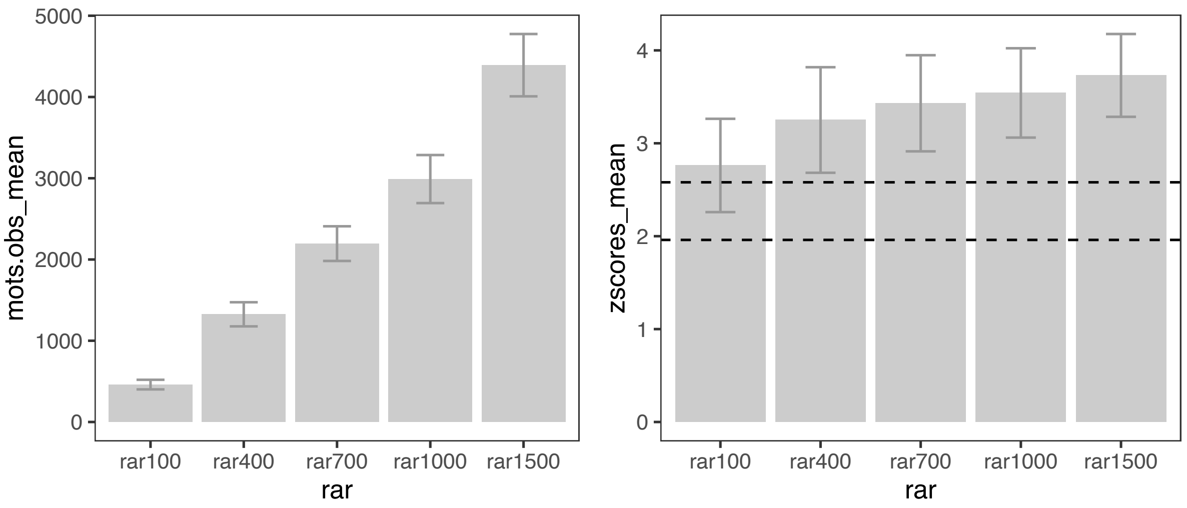

Table S1 | Plant identity of autotrophic (mutualistic) and mycoheterotrophic (antagonistic) plants from this study. In total, we collected 220 autotrophic and 60 mycoheterotrophic root tips samples. For the autotrophic plants, 123 samples could be identified by DNA sequencing; these are listed here. 99 samples among 28 autotrophic, and 45 samples among the five mycoheterotrophic plant species had Glomeromycotina reads. After removing samples with less than 100 reads, and only considering an OTU present in a sample when represented by at least 5 reads, we obtained a total of 77 samples among 21 autotrophic species, and 27 samples among the five mycoheterotrophic species. Rows in grey are excluded taxa due to the absence of Glomeromycotina reads; rows in orange are taxa excluded from subsequent analyses because overall at species level, they had < 500 reads (total number of reads in autotrophic plants in the table excludes the taxa in orange).

Mutualistic Plants	Life form	sequenced samples	samples with AM fungi	excluding samples (< 100 reads)	AM fungi reads
Acacia	tree	1	1	1	6350
Araceae	herb	3	3	3	10456
Araliaceae	shrub	3	2	2	7122
Arecaceae	palm	1	0	0	0
Aspidosperma	tree	23	20	13	13667
Bromeliaceae	herb	1	0	0	0
Clusia	tree	4	4	4	43344
Cupania	tree	2	2	0	126
Dicorynia	tree	4	2	2	2302
Dilleniaceae	tree	1	1	1	2819
Eperua	tree	12	12	11	11483
Fabaceae2	tree*	2	2	2	5771
Fabaceae1	tree**	4	3	3	4441
Hydrochorea	shrub	1	0	0	0
Ilex	tree	1	1	1	844
Inga	tree	3	1	1	19099
Iryanthera	tree	1	0	0	0
Malphighiaceae	vine	12	11	9	20624
Metteniusaceae	tree	7	5	5	4616
Mimosa	shrub	1	1	1	3486
Morus	tree	3	3	3	2821
Myristica	tree	1	1	0	0
Odontadenia	liana	1	1	0	0
Paloue	tree	8	6	6	11635
Peltophorum	tree	1	1	0	401
Protium	tree	5	4	4	6104
Sapindaceae	tree	4	1	1	1285
Sapotaceae	shrub	3	2	1	246
Schizeaeceae	fern	1	1	0	0
Schradera	liana	2	2	2	1882
Tapirira	tree	6	5	1	5124
Urticaceae	tree	1	1	0	293
**Total**		**123**	**99**	**77**	**185275**
**Antagonistic Plants**					
Soridium spruceanum	herb	9	8	4	15290
Dictyostega orobanchoides	herb	7	5	3	5132
Voyria aphylla	herb	3	3	3	8559
Voyriella parviflora	herb	34	22	10	3357
Gymnosiphon breviflorus	herb	7	7	7	148522
**Total**		**60**	**45**	**27**	**180860**

Identification of life form of these plants is based on the life form of their best Blast hit:
*best Blast hit (95.9%) is *Martiodendron mediterraneum* (does not occur in the region)
** best Blast hit (99.6%) is *Acaciella chamalensis* / *Enterolobium cyclocarpum* (does not occur in the region).

References

Jacquemyn H, Brys R, Merckx VSFT, Waud M, Lievens B, Wiegand T. 2014. Coexisting orchid species have distinct mycorrhizal communities and display strong spatial segregation. *New Phytologist* **202**: 616-627.

Lozupone C, Knight R. 2005. UniFrac: a new phylogenetic method for comparing microbial communities. Applied and Environmental Microbiology **71**:8228-8235.

Oksanen J, Blanchet FG, Kindt R, Legendre P, Minchin PR, O'Hara RB, Simpson GL, Solymos P, Stevens MHH, Wagner H. **2015**. vegan: Community Ecology Package. R package version 2.3-1. *http://CRAN.R-project.org/package=vegan*.
